# Neural Signaling of Food Healthiness Associated with Emotion Processing

**DOI:** 10.3389/fnagi.2016.00016

**Published:** 2016-02-10

**Authors:** Uwe Herwig, Matthias Dhum, Anna Hittmeyer, Sarah Opialla, Sigrid Scherpiet, Carmen Keller, Annette B. Brühl, Michael Siegrist

**Affiliations:** ^1^Clinic for Psychiatry, Psychotherapy and Psychosomatics, University Hospital of Psychiatry in ZürichZürich, Switzerland; ^2^Department of Consumer Behaviour, Institute for Environmental Decisions, ETH ZürichZürich, Switzerland; ^3^Behavioural and Clinical Neuroscience, Institute and Department of Psychiatry, University of CambridgeCambridge, UK

**Keywords:** functional neuroimaging, food, healthiness, amygdala, midbrain, gender

## Abstract

The ability to differentiate healthy from unhealthy foods is important in order to promote good health. Food, however, may have an emotional connotation, which could be inversely related to healthiness. The neurobiological background of differentiating healthy and unhealthy food and its relations to emotion processing are not yet well understood. We addressed the neural activations, particularly considering the single subject level, when one evaluates a food item to be of a higher, compared to a lower grade of healthiness with a particular view on emotion processing brain regions. Thirty-seven healthy subjects underwent functional magnetic resonance imaging while evaluating the healthiness of food presented as photographs with a subsequent rating on a visual analog scale. We compared individual evaluations of high and low healthiness of food items and also considered gender differences. We found increased activation when food was evaluated to be healthy in the left dorsolateral prefrontal cortex and precuneus in whole brain analyses. In ROI analyses, perceived and rated higher healthiness was associated with lower amygdala activity and higher ventral striatal and orbitofrontal cortex activity. Females exerted a higher activation in midbrain areas when rating food items as being healthy. Our results underline the close relationship between food and emotion processing, which makes sense considering evolutionary aspects. Actively evaluating and deciding whether food is healthy is accompanied by neural signaling associated with reward and self-relevance, which could promote salutary nutrition behavior. The involved brain regions may be amenable to mechanisms of emotion regulation in the context of psychotherapeutic regulation of food intake.

## Introduction

The ability to perceive food as being advantageous or disadvantageous to one’s own health, and guiding one’s nutritional behavior accordingly, promotes good health. This is particularly important considering that the burden and the economic impact of nutrition related medical conditions and unhealthy lifestyles is high (Striegel-Moore and Bulik, 2007; Hoek and King, 2008). Nutrition, or food intake, may trigger, or may cause pleasant and unpleasant feelings, and can such be considered to be associated with the activation of brain regions that process emotions (Rolls, 2005, 2008; Siep et al., 2009; Ziauddeen et al., 2012; Meye and Adan, 2014; Morton et al., 2014). A feature of tasty, but unhealthy nutrition consists of appetitive emotions occurring with impaired impulse control and self-guiding related to respective food stimuli, despite knowledge of a possible disadvantageous health value (Glanz et al., 1998). For example, eating chocolate is accompanied by a positive emotion, whereby resisting eating an offered chocolate may result in unpleasantness and may require impulse control. This unpleasantness signal makes sense from an evolutionary point of view, as the ingestion of high caloric food is advantageous for an organism’s survival. However, many people, for instance those with obesity, diabetes or other nutrition related conditions, have to control eating certain foods that they desire. Evaluating and choosing healthy food on an individual and situation based level in this way is especially important.

In everyday life, many people do not actively reflect on whether the food they eat is healthy, but more so on whether it is tasty (Glanz et al., 1998). However, consciously reflecting about the healthiness of a food item can influence eating behavior. Identifying the brain regions involved in the evaluation of food healthiness might help to understand which cognitive strategies are utilized to promote salutary nutrition. We investigated brain activity associated with single subject’s conscious food healthiness evaluation and rating and focused on brain regions known to be involved in emotion processing and regulation. We were interested in which brain areas signal differentially when evaluating a food item to be of higher healthiness compared to lower healthiness.

Earlier studies on the neural processing of the visual presentation of food stimuli in healthy subjects showed heightened activation in insular and orbitofrontal cortex (OFC) regions, when hungry (Porubská et al., 2006) and not hungry (Killgore et al., 2003; Simmons et al., 2005). These results are consistent with reports of gustatory representation in these areas (Rolls, 2001; Kringelbach, 2004). Furthermore, the OFC has been shown to integrate different sensory modalities such as gustation and olfaction (Rolls, 2001). Fuhrer et al. (2008) analyzed brain activity during the presentation of food items compared to other stimuli. They found stronger activation of the medial prefrontal, insular, anterior cingulate, and striatal regions when participants were presented with food stimuli. Siep et al. (2009) reported activity in reward-associated brain regions, the amygdala and OFC, when assessing high versus low caloric nutrition. Rolls (2008) discussed a central involvement of the orbitofrontal and anterior cingulate cortex in the multimodal representation of food particularly associated with reward. These suggestions were also supported by Frank et al. (2010). Medial and lateral prefrontal cortex areas are also involved in value based decision making (Deco et al., 2013; Dixon and Christoff, 2014), which was also required in our task. Following these findings, regions of interest to be considered in our study were the medial and dorsolateral prefrontal cortex regions, anterior/subgenual cingulate gyrus, OFC, anterior insula, amygdala, ventral striatum, medial thalamus, and midbrain.

In previous studies that have investigated neural activation associated with food healthiness, food healthiness processing in general was related to cognitive domains such as attention (e.g., Hare et al., 2011; Grabenhorst et al., 2013), but signaling of healthiness versus non-healthiness in distinct brain areas was not addressed and the individual estimation of healthiness evaluation was not considered (e.g., Frank et al., 2010; Killgore and Yurgelun-Todd, 2010). The novelty of our study relies on (i) the direct comparison of brain activation associated with high and low health value in healthy subjects, and (ii) on the investigation of the single subject level of food healthiness evaluation. Therefore, our analysis was not based on a general a priori categorization of the food items into healthy and unhealthy categories, but on the single subject rating of each food item concerning perceived health value. Given that the subjective valence of different foods can vary, we individually determined the grade of healthiness related to each presented food item and considered the individual results for the analysis. As it was previously shown that males and females may differ regarding their estimation of healthiness of nutrition and other food related aspects (Killgore and Yurgelun-Todd, 2010; Geliebter et al., 2013), we further aimed to identify gender differences at the level of neural activation concerning food evaluation. We expected the differential evaluation of food healthiness to be associated with the activation of brain areas related to emotion processing, especially in more primordial brain regions such as the midbrain, amygdala and ventral striatum regarding an emotional connotation, and in the insula and OFC regarding viscero-sensitive interoception. A cognitive approach, however, would involve higher cortical regions such as medial prefrontal and dorsolateral prefrontal cortex regions.

## Materials and Methods

### Ethical Statement

The study was approved by the Kantonale Ethikkommission Zuerich, Switzerland (as stated in the submission questionnaire).

### Subjects

Forty-one healthy subjects (age 20–46 years, mean 24.8, SD 4.6; all right handed; 22 males, BMI mean 22.9, SD 2.4, range 19.9–28.6 with n = 1 > 26; 19 females, BMI mean 21.3, SD 2.1, range 18.0–24.6; none with dietary needs, 39 with academic background, mostly students, 2 medical assistance professionals) were recruited to participate in this study and gave written informed consent. The study was approved by the local ethics committee. Further, the subjects were neither hungry throughout scanning, nor had they had a major meal within an hour prior scanning. Four subjects were excluded afterward because of sudden movement artifacts (exceeding more than 3 mm in at least one direction) or other technical reasons, so that the data of 37 subjects (age 20–46, mean 24.9, all right handed, 19 males, 18 females; **Table 1**) were analyzed. The subjects were healthy (assessed with clinical interview based on ICD-10 and DSM-IV) and did not take any psychotropic medication or have any psychiatric, neurological, or other relevant medical history that would affect the results of this study. We also assessed self-ratings of depression (SDS, German version; Zung, 2005) and state-trait anxiety inventory (STAI) to control for affective or anxiety symptoms (**Table 1**).

**Table 1:** Demographic and psychometric data of the subjects.

	Total mean	Males mean	Females mean	Males vs. females
	(*SD*, range)	(*SD*, range)	(*SD*, range)	*p*
Subjects (*n*)	37	19	18	
Age (years)	24.8 (4.6, 20–46)	24.4 (4.0, 20–36)	25.3 (5.5, 20–46)	0.58
SDS	45.6 (3.8, 36–53)	45.2 (1.9, 42–48)	46.1 (4.2, 36–53)	0.48
STAI				
- State	43.6 (2.8, 38–49)	43.4 (2.9, 38–49)	43.8 (2.7, 39–48)	0.71
- Trait	43.7 (4.3, 37–56)	43.0 (4.2, 37–52)	44.4 (4.2, 39–56)	0.32


*SDS, Self-ratings of depression; STAI, state-trait anxiety inventory.*

### Experimental Design

**FIGURE 1:**
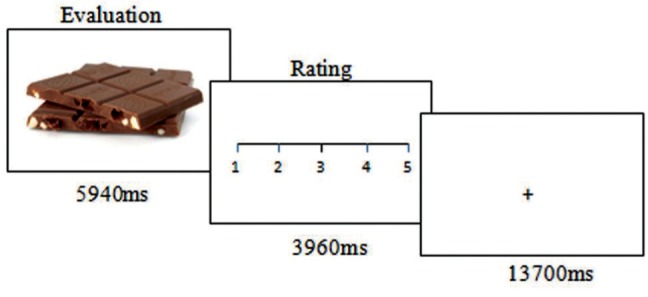
**Experimental task.** The trials started with displaying a food photograph for nearly 6 s while the participants evaluated the healthiness of the food item. This was followed by a rating period of nearly 4 s. A baseline condition of nearly 16 s was implemented between trials.

During fMRI scanning, the subjects evaluated the healthiness of different food items presented in photographs. The photographs showed the food items on a white background, in such a way that only the food item was visible (examples in **Figure 1**). The food items were divided into two halves representing more or less healthy or unhealthy food, respectively. The food photographs were presented for 5940 ms (equivalent to three repetition times, TR, for the fMRI volumes). In this “evaluation” period, the subjects were instructed to look at the photo and to estimate the healthiness of the respective food item. Subsequently, a visual analog scale ranging from 1 to 5 was presented for 3960 ms (two volumes) on which the subjects indicated the individually estimated nutrition value between very healthy (5) to very unhealthy (1) by moving a cursor using a trackball with the right hand (**Figure 1**), the “rating period”. Altogether, 40 food stimuli were presented in a randomized order. The following baseline period (13700 ms, 7 TR) was of sufficient duration to allow the blood oxygen level-dependent signal to wear off before the next trial. The task was programmed with Presentation^TM^ (Neurobehavioral Systems, USA) and presented via digital video goggles (Resonance Technologies, Northridge, CA, USA). Photographs were sized to fill approximately two thirds of the screen diameter, so that the food item could have been identified immediately with minimal eye movements required. After scanning, the subjects were asked to rate the healthiness of the food items again and also the grade of subjective tastiness, from very tasty (5) to not tasty at all (1), on visual analog scales.

We specifically assessed the difference in healthiness evaluation within the sample of food pictures on a single subject level. As such, we explicitly compared the items as being of high or low healthiness as they were rated by the individual. In order to better discriminate healthy ratings from unhealthy ones, we separated both groups by a group of stimuli with intermediate healthiness (group definition below).

### Data Acquisition

Imaging was performed with a 3.0 T GE Signa^TM^ HD Scanner (GE Medical Systems, Milwaukee, WI, USA). Echo planar imaging was performed for fMRI (repetition time TR/echo time TE 1980 ms/32 ms, 22 sequential axial slices, whole brain, slice thickness 3.5 mm, 1 mm gap, resulting voxel size 3.125 mm × 3.125 mm × 4.5 mm, matrix 64 × 64 pixels, field of view 200 mm, flip angle 70°). 528 volumes were obtained per subject, 12 per trial. Four initial volumes were discarded to allow for equilibration effects, seven volumes were added for a final baseline. High-resolution 3-D T1 weighted anatomical volumes were acquired (TR/TE 9.9/2.9 ms; matrix size 256 × 256; 1 mm × 1 mm × 1 mm resolution) for co-registration with the functional data.

### Data Analysis

FMRI data were analyzed using BrainVoyager^TM^ QX 2.0 (Brain Innovation, Maastricht, The Netherlands). Pre-processing of the functional scans included motion correction, slice scan time correction, high frequency temporal filtering, and removal of linear trends. Functional images were superimposed on the 2D anatomical images and incorporated into volume time courses. The individual volume time courses were transformed into Talairach space resulting in a voxel size of 3 mm × 3 mm × 3 mm and then spatially smoothed with an 8 mm Gaussian kernel for subsequent group analysis. From each included subject (*n* = 37), the individual food healthiness evaluation periods of each single food item presentation were considered. We pre-defined three categories of healthiness ratings: high, medium, and low. The categories were mathematically divided considering the highest and lowest 1.5 score periods on the scale for the analysis of high and low healthiness, respectively. Thus, low healthiness was defined between 1.00 and 2.50, medium healthiness between 2.51 and 3.49, and high healthiness between 3.50 and 5.00, based on the distribution of the evaluation ratings. Individual experimental design matrices for each subject for the fMRI-analysis were built comprising the individually rated items meeting the three conditions (low, medium, high healthiness) and the respective three conditions with presentation of the rating scale as predictors, resulting in six predictors for the design matrix. The periods were modeled as epochs using a two-gamma hemodynamic response function provided by BrainVoyager^TM^ and were adapted to the applied period duration.

The fMRI data analysis, based on the general linear model (GLM), comprised the following steps: First, fixed effects analyses were calculated separately for each subject for the contrasts comparing the individual conditions of evaluation and rating of ‘high healthiness’ versus ‘low healthiness’, resulting in summary images. The summary images were subjected to second level group analyses. Thus, those trials in which the food photographs were rated as ‘high’ and ‘low’ healthiness were considered for contrast analysis. The ‘medium’ rated items were also modeled as a condition in the analysis protocol but not considered for the final fMRI analysis and served therefore as a “buffer” between ‘low’ and ‘high’ for better discrimination. We further differentiated between the evaluation and the rating period. The evaluation period was the primary period of interest with the pure mental act of reflecting about and estimating the healthiness of the presented food item without a motor command or other distracting activity. The rating period was the period of giving feedback concerning the healthiness estimation. Both, evaluation and rating period, were functionally and timely coupled, nevertheless, we decided on a separated analysis. To analyze the evaluation and rating periods, three-dimensional statistical parametric maps were calculated for the groups using a random effects analysis.

Because of the approach with individual data of each subject for each food item, the data were not suitable for a continuous or regression analysis, which would have been suitable for a mean value derived from all subjects for the single food items.

The main analysis therefore focused on the contrasts “evaluation high healthiness > evaluation low healthiness” (e-hi > e-lo) and “rating high healthiness > rating low healthiness” (r-hi > r-lo). The voxel-wise threshold for reporting results in the random effects analysis was set at *p* < 0.005. To correct for multiple comparisons, a Monte Carlo simulation was used (Goebel et al., 2006) for estimating cluster-level false-positive rates on these maps, yielding after 10.000 iterations a minimum cluster size threshold of 10 voxels of 3 mm × 3 mm × 3 mm (270 mm^3^), corresponding to a corrected cluster level *p* < 0.02.

We also assessed the brain activity associated with food healthiness evaluation and rating in predefined anatomical cubic ROIs, which are known to be related to emotion processing. For the larger cortical regions of the insula, subgenual (sg)ACC, OFC, DLPFC, and DMPFC, ROIs were constructed using 4 × 4 × 4 functional voxels (edge length 12 mm × 12 mm × 12 mm, volume 1728 mm^3^ each). The ROIs were placed according to the Talairach Client (Lancaster et al., 2000) and prior studies: DMPFC *x* = 6/–6, *y* = 6, *z* = 50, covering Brodmann Area (BA) 6 and 8 in the superior frontal gyrus; and DLPFC *x* = 43/–43, *y* = 18, *z* = 30, covering mainly BA 9 in the middle frontal gyrus (Northoff et al., 2006; Herwig et al., 2010, 2011); anterior insula *x* = 33/–33, *y* = 16, *z* = –1 (Craig, 2009; Paulus and Stein, 2010); ventral striatum (10/–10, 6, –6; McClure et al., 2004; Heimer and Van Hoesen, 2006), amygdala (Costafreda et al., 2008, edge length 6 mm, 22/–22, –6, –12), medial thalamus (0, –12, 4), midbrain (0, –23, –12), OFC (0, 52, –1), ACC (0, 38, 1), sgACC (0, 17, –9).

Finally, in order to assess the influence of gender on the food evaluation and food rating periods, we introduced this variable as a covariate in a further analysis using the same statistical approach and thresholds.

## Results

### Behavioral Data

Thirty-seven subjects were included in the analysis (demographic data including normal anxiety and depressiveness ratings in **Table 1**). The subjects attributed high healthiness to 16.2 items (SD 3.0), medium healthiness to 5.7 items (SD 3.1), and low healthiness to 18.0 items (SD 2.1), on average. This resulted in *n* = 601 trials with high healthiness, *n* = 212 trials with medium healthiness, and *n* = 667 trials with low healthiness, overall. Correlating healthiness with tastiness in the groups of pictures that were rated as healthy and in the group rated as unhealthy did not reveal any significant results: healthy/taste *r* = –0.06 (mean healthy food pictures 4.4, taste health group 4.1), unhealthy/taste *r* = –0.11 (mean health rating in the unhealthy food pictures 1.8, mean taste in that group 3.6). However, when correlating the grades of healthiness and tastiness in the whole group, we found a positive correlation (*r* = 0.52), meaning that healthier food items were also rated as tastier.

### fMRI Results

We performed whole brain analyses on the contrasts “evaluation high healthiness > evaluation low healthiness” (e-hi > e-lo) and “rating high healthiness > rating low healthiness” (r-hi > r-lo).

**FIGURE 2:**
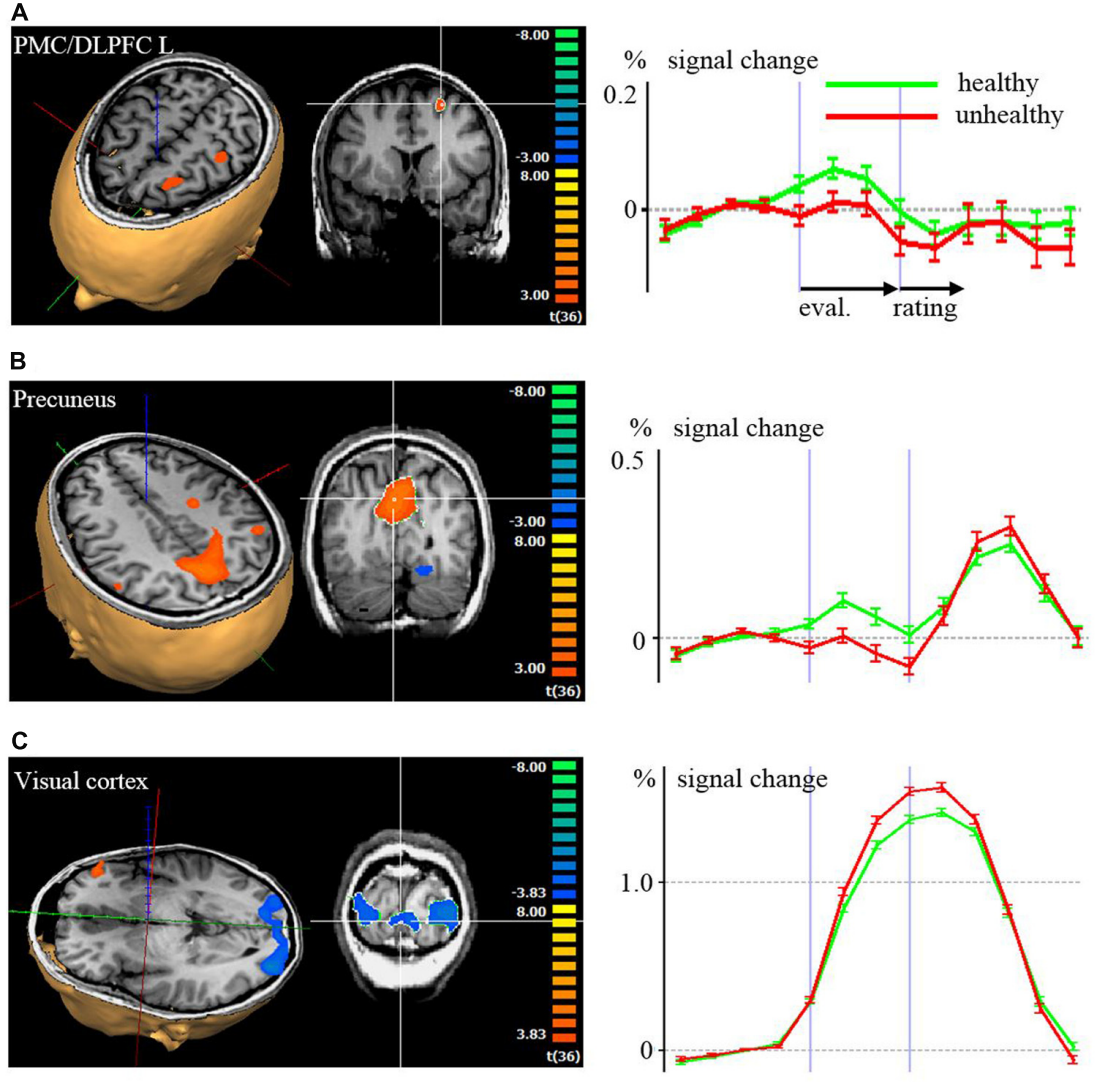
**Brain activation with color coded maps and time courses with signal changes according to a random effects analysis (p < 0.001) of the evaluation (eval.) and rating period, comparing high healthiness against low healthiness: (A) left dorsolateral prefrontal cortex (DLPFC), (B) precuneus, (C) visual cortex.** In the 3D visualizations, red, blue, and green axes indicate the coordinate system. In the 2D coronal slice, the region with statistical significant activation of which the time course is derived is marked with the white crosshair. In the time course diagrams on the left side, the first vertical violet bars after the y-axis correspond to the activations during the first volume of the evaluation period. The second vertical violet bars correspond to the activations during the first volume of the rating period.

**FIGURE 3:**
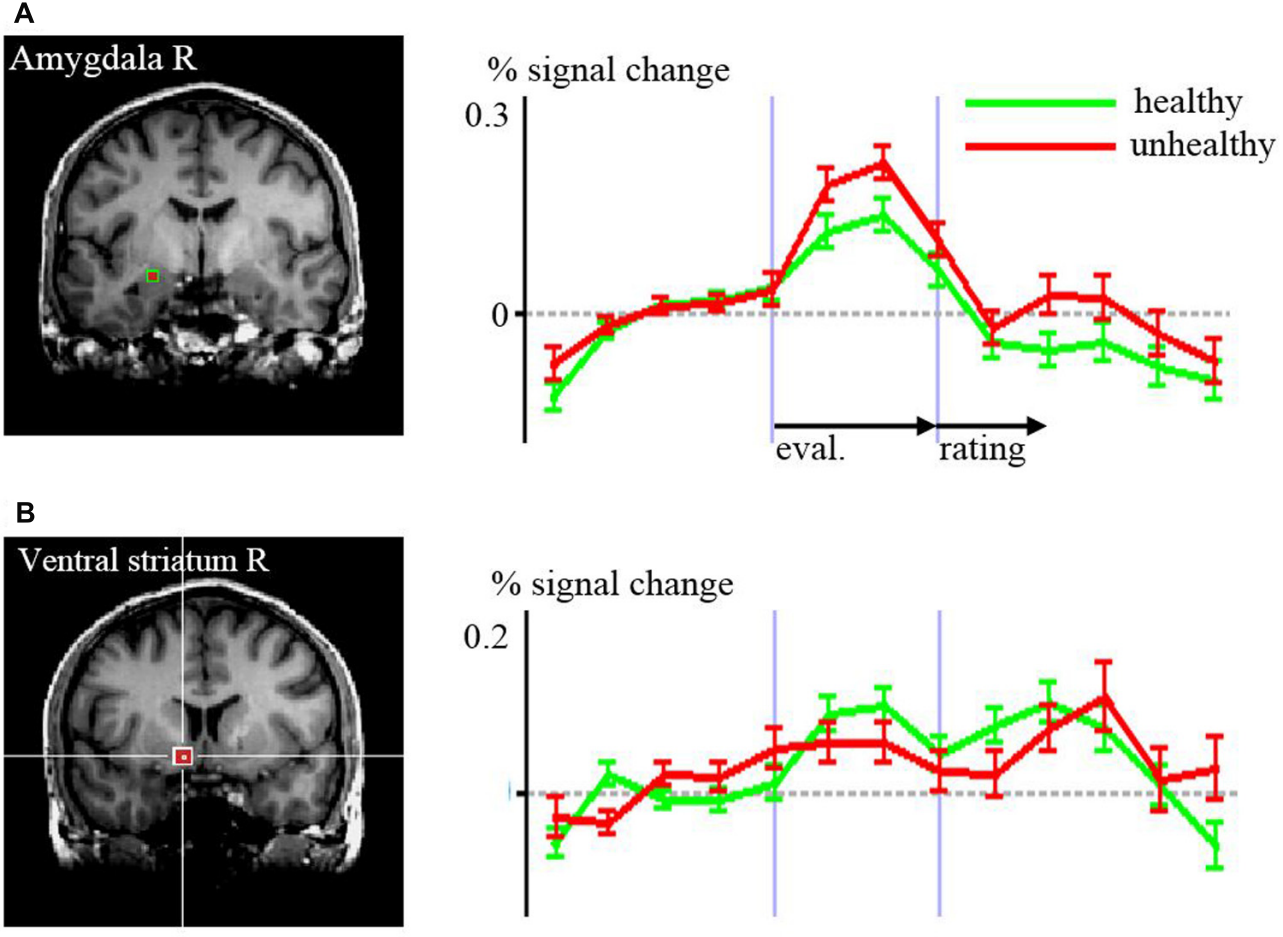
**Time courses of signal changes according to the region of interest analyses of healthy versus unhealthy food in (A) amygdala, (B) ventral striatum.** The regions are indicated by the red squares in the structural MR images Differences in the time courses were significant for the evaluation (eval.) period in the amygdala (*p* = 0.048) and for the rating period in the ventral striatum (*p* = 0.005).

Regarding the evaluation period, we found higher activation in a left superior/medial prefrontal cortex region covering BA 6, 8, 9, [premotor cortex (PMC) and DLPFC, **Figure 2A**] and in precuneus and lateral parietal cortex regions associated with the evaluation of food pictures subjectively rated as high in healthiness. Higher activation in primary and associative visual cortex was associated with the low healthiness food pictures (**Table 2**; **Figure 2**). The ROI analysis was used in order to assess activation in emotion processing related brain areas, and revealed higher activity in the right amygdala associated with evaluation of unhealthy food stimuli compared to healthy stimuli (*p* = 0.048, *t* = –2.05). Both evaluations (healthy and unhealthy) activated the amygdala compared to baseline (right and left *p* < 0.00001, *t* > 7; **Figure 3**).

**Table 2:** Contrast evaluation period of healthy vs. non-healthy food photographs in the whole brain analysis.

	BA	Peak *x*	Peak *y*	Peak *z*	*t*	*P*	mm^3^
PMC/DLPFC L	6, 8, 9	–18	8	49	3.73	0.0007	766
IFG R	11, 44, 45	42	44	–8	4.96	0.0000	6832
Posterior insula R	13	36	–37	22	3.63	0.0009	521
Superior temporal ctx	22	36	–40	1	3.46	0.0014	649
Precuneus	7	–3	–70	34	4.75	0.0000	16393
Occipital ctx	18	–30	–88	–2	–5.41	0.0000	31725
Postcentral gyrus L	3	–33	–28	52	3.55	0.0011	472
Temporo-occipital ctx L	22, 39	–39	–52	16	4.84	0.0000	8614
Temporo-parietal ctx R	39	45	–58	28	4.50	0.0001	5269


*PMC, premotor cortex; DLPFC, dorsolateral prefrontal cortex; IFG, inferior frontal gyrus ctx cortex; BA, Brodmann Area; R, right; L, left; SD, standard deviation.*

Regarding the rating period, right ventral striatal activity was stronger when rating food as being healthy compared to unhealthy (*p* = 0.008, *t* = 2.82). DLPFC BA 46 was bilaterally more active with the rating of unhealthy food compared to healthy (right *p* = 0.020, *t* = –2.44; left *p* = 0.041, *t* = –2.12).

**FIGURE 4:**
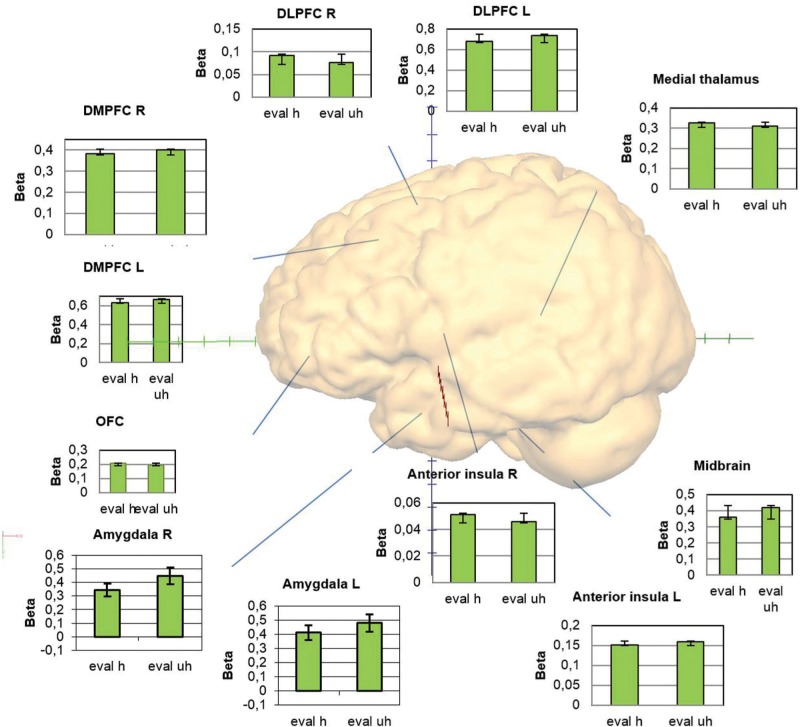
**Overview of the activations within the regions of interest providing graphs with the beta weights for the conditions evaluation healthy and unhealthy**.

Food healthiness evaluation and rating were both associated with significant neural activation in the other ROIs such as the MPFC, OFC, ACC, insula (apart for right anterior insula and evaluation), medial thalamus and midbrain, but without differences in regard to subjective healthiness (**Figure 4**; **Table 3**).

**Table 3:** Results of the comparisons of the conditions healthy and unhealthy in different regions of interest providing statistical *p*- and *t*-values for each, the evaluation (Eval) and the rating (Rat) periods.

Anatomic	Evaluation	Evaluation	Evaluation	Rating	Rating	Rating
region	healthy >	healthy	unhealthy	healthy >	healthy	unhealthy
	unhealthy			unhealthy		
Tal. *X/Y/Z*	*p*/*t*	*p*/*t*	*p*/*t*	*p*/*t*	*p*/*t*	*p*/*t*
Amygdala R						
22/–6/–12	0.048/–2.05	<0.0001/7.26	<0.0001/7.16	0.54/–0.62	0.53/–0.64	0.93/–0.09
Amygdala L						
–22, –6, –12	0.14/–1.50	<0.00001/6.92	>0.00001/6.01	0.85/0.19	0.63/0.49	0.71/0.37
Ventral striatum R						
10/6/–6	0.083/–0.25	0.001/3.64	0.005/3.03	0.005/3.00	0.001/3.71	0.08/–1.81
Ventral striatum L						
–10/6/–6	0.48/–0.75	0.0002/4.12	0.0003/4.03	0.30/–1.05	<0.00001/5.74	<0.00001/6.12
DMPFC R						
6/6/50	0.73/–0.342	<0.00001/5.005	<0.00001/5.27	0.94/0.071	<0.00001/22.23	<0.00001/17.14
DMPFC L						
–6/6/50	0.51/–0.66	<0.00001/8.77	<0.00001/8.59	0.56/–0.59	<0.00001/19.11	<0.00001/17.16
DLPFC R						
43 / 18 / 30	0.77/0.30	0.20/1.28	0.34/0.96	0.020/–2.44	<0.00001/9.18	<0.00001/9.62
DLPFC L						
–43/18/30	0.34/–0.98	<0.00001/9.60	<0.00001/10.04	0.036/–2.17	<0.00001/8.58	<0.00001/9.48
Anterior cingulated						
0/38/1	0.079/–1.81	<0.00001/9.26	<0.00001/9.32	0.38/–0.89	<0.00001/13.69	<0.00001/12.20
Subgenual cingulated						
0/17/9	0.29/–1.08	0.00015/4.28	0.00002/4.91	0.59/0.54	0.036/–2.18	0.027/–2.31
Anterior insula R –33/16/–1	0.94/0.07	0.37/0.90	0.39/0.86	0.37/0.90	<0.00001/10.48	<0.00001/9.19
Anterior insula L 33/16/–1	0.90/–0.13	0.039/2.14	0.030/2.26	0.73/0.35	<0.00001/9.98	<0.00001/9.16
Medial thalamus						
0/–12/4	0.74/0.33	<0.00001/5.23	<0.00001/4.40	0.79/0.27	<0.00001/14.57	<0.00001/11.28
Midbrain						
0/23/–12	0.19/–1.32	<0.00001/8.06	<0.00001/7.95	0.71/–0.37	<0.00001/11.17	<0.00001/11.9
Orbitofrontal cortex						
3/49/–12	0.80/0.25	0.00015/4.23	0.0036/3.12	0.0056/2.97	< 0.00001/–6.00	<0.00001/–8.03


*The *x, y, z* coordinates correspond to the centers of the named regions.*

**FIGURE 5:**
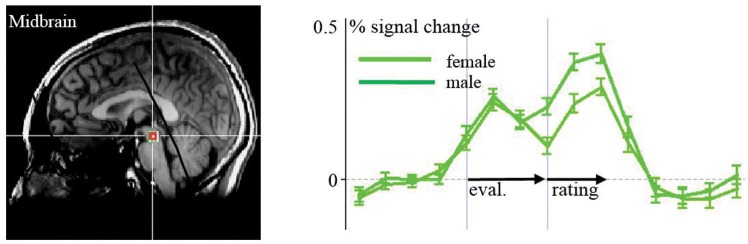
**ROI-Analyses of females vs. males: activation in the midbrain to healthy food items is stronger in females than in males**.

When assessing differences between males and females in the ROI analysis, we found higher activity in the right ventral striatum among males compared to females in the perception and evaluation of unhealthy stimuli (*m* > *f*; *p* = 0.008, *t* = 2.813). This contrast was at the borderline to significance in the medial thalamus (*p* = 0.058, *t* = 1.959). Activation in the midbrain region was higher among females associated with the rating period of healthy stimuli compared to unhealthy stimuli (*p* = 0.028, *t* = 2.293; **Figure 5**). We found no differences between males and females in the amygdala, insula, and prefrontal regions.

## Discussion

Our main interest was on neural signaling associated with evaluating higher versus lower healthiness of food stimuli on a single subject level. We found an association of higher healthiness evaluation with higher activity of the PMC/DLPFC BA 6/8/9 and in the precuneus in the whole brain analysis and in the ROI analysis of the rating period in the ventral striatum and the OFC. Contrary to our hypothesis, we found an association of lower food healthiness with higher activation in the amygdala,. This was also found bilaterally in more anterior and lateral regions of the DLPFC (BA 46) as well as in primary and secondary visual areas. Nearly all other areas known to be involved in a network of brain areas associated with emotion processing, such as the MPFC, OFC, ACC, insula, medial thalamus, and midbrain, were activated generally when associated with the act of food healthiness evaluation and/or respective rating, but not specifically for healthy or unhealthy, and of course within the frame of our study without exclusion of other cognitive components that may be attributed to the activation. However, this at least implicates a relationship between the estimation of nutritional healthiness and emotional signaling in distinct emotion processing areas. The only gender difference was reflected by a higher activation in the midbrain of females associated with healthy stimuli.

### Food Health Evaluation and Emotion Processing

The general association between nutrition and emotion has been proven by multiple studies (Rolls, 2005, 2008; Siep et al., 2009; and many others). Denton et al. (2009) have shown hunger and satiation as belonging to “primordial emotions” deeply rooted in central-nervous information processing and developed early in evolution. From an evolutionary point of view, one can even suggest that the complex system of emotions may at least in part originate from the bodily signaling associated with the ingestion of food and its evaluation in early species throughout evolution (Denton et al., 2009). Getting nutritious food is necessary for survival and associated with feelings of reward. Conversely, being deprived of food is unpleasant and potentially life threatening. As such, one may argue that neural emotion processing might have evolved at least in part from nutrition related neural processing. Considering the long term consequences of healthiness and favoring them over the short term benefits of tasty but unhealthy food, however, is often difficult and requires awareness for health aspects and self-control (Liberman and Trope, 2008; Hare et al., 2011).

According to our findings, regions associated with a differential healthiness signal comprise the DLPFC, MPFC, precuneus, amygdala, and ventral striatum. The PMC/DLPFC region showed stronger activation in more posterior and superior regions (BA 6/8/9) associated with the rating of higher healthiness, and in more rostral regions (BA 46) associated with the rating of lower healthiness. Hare et al. (2011) reported the lateral prefrontal cortex in BA 8/9 and 46/47 showed increased activity during a condition where subjects were asked to generally consider the healthiness of a food item. Further, BA 9 of the DLPFC appeared to modulate ventromedial PFC to promote health information (Hare et al., 2009, 2011). In a study assessing brain activation during the regulation of the desire for food intake by using reappraisal strategies, the DLPFC, as well as medial and inferior frontal PFC areas, were activated. This suggests that these areas have an impact in controlling food intake (Hollmann et al., 2012). The identified regions of the DLPFC, which is generally known to be involved in cognitive and executive control (e.g., Disner et al., 2011; Fuster, 2000), are suggested to be involved centrally in guiding nutrition relevant evaluation and behavior. The anterior and subgenual cingulate cortices, involved in conflict detection (Carter and van Veen, 2007), were not activated specifically with evaluation of healthy or unhealthy food.

Important concomitant signals for evaluations of food may arise from the amygdala and ventral striatum. However, contrary to what we hypothesized, the amygdala in our study exerted a stronger signal associated with lower healthiness and the ventral striatum signaled higher healthiness. Both regions are known to be tightly coupled with emotion processing. The ventral striatum is part of the reward system (Salamone and Correa, 2012) and the amygdalae are central processors of emotional signals (Costafreda et al., 2008; Pessoa and Adolphs, 2010). In the context of food processing, Grabenhorst et al. (2013) found that nutritional information biased food evaluations in the amygdala, potentially reflecting an active amygdala participation in food choice. Siep et al. (2009) also reported the amygdala as being active when attending and evaluating food. However, when simply watching food, the amygdala was reported as inactive (Siep et al., 2009; Frank et al., 2010). This underlines the role of the amygdala in evaluation and, possibly, choice or “what if” processes. It may provide bottom–up signaling of the grade of healthiness and, as our findings imply, “warn” by a higher activation when an unhealthy food item is detected. Furthermore, amygdala signaling may be associated with the retrieval of autobiographic and episodic content concerning food, biasing approach or avoidance behavior. On the other hand, the amygdala is a central recipient of cognitive control processes (e.g., Ochsner and Gross, 2005; Herwig et al., 2007) and thus is susceptible to deliberate regulation of food choice and consumption. In that context, “warning” signals can also be intentionally ignored or suppressed when deciding to eat unhealthy food.

The ventral striatum was consistently activated during evaluation and rating bilaterally, and showed right-sided higher activity during the rating of higher healthiness. This may be interpreted as a reward signaling, since the ventral striatum is centrally involved in the brain reward system (Hollmann et al., 2012; Kringelbach et al., 2012). Earlier studies reported, however, that salutary food might be regarded as less tasty than unhealthier food items (Glanz et al., 1998), because healthy food might cause less reward signaling than unhealthy food. We found a positive correlation between health and taste rating, which might result from a bias due to actively making oneself aware of the health value, which promotes a reward signal in the case of healthy foods. It has been shown that food and drug cues activate the same reward related brain regions (Tang et al., 2012). The cognitive act of intentionally reflecting on the healthiness of food prior to the concrete selection of nutrition, which is often not regularly done, might lead to preferring healthy food, as biased by a reward signal in the context of health evaluation. This might be a simple cognitive strategy for healthier nutrition and better impulse control compared to every-day nutrition without actively reflecting on health value.

The OFC is assumed to be involved in the representation of emotional value linked to reward and decision-making, thereby guiding behavior (Kringelbach, 2005; Schoenbaum et al., 2011). In our case, the OFC was more strongly activated when rating healthy food than when rating unhealthy food. This supports an association between health evaluation and reward in our context. We expected insular regions to be differentially activated by healthiness, but despite a bilaterally prominent general activation during the rating period, no specific healthiness signal was detected. Nevertheless, the strong activation reflects its involvement in associated interoceptive awareness processes (Critchley et al., 2004; Paulus and Stein, 2010). Finally, the unhealthy food items activated the areas within the primary and associative visual cortex more strongly. Whether this may be due to neural processing related to the unhealthiness or to basic visual aspects remains open.

A potential clinical application could be the utilization of cognitive regulation strategies such as reappraisal in order to control food intake when needed (Siep et al., 2012; Yokum and Stice, 2013). A recent reappraisal study for instance supported applying the strategy of reflecting about the long-term benefits of not eating (Yokum and Stice, 2013). Incorporating health aspects in such strategies may advance the application within psychotherapeutic control of eating behavior.

### Nutrition and Self-Related Brain Activation

Another interesting finding was the prominent activation of the precuneus, particularly its cognitive self-representation related domain, associated with health evaluation. In an earlier study, participants had estimated the risk of certain hazards presented as verbal terms. It was found that the precuneus was activated when evaluating a higher risk (Herwig et al., 2011). The precuneus is regarded evolutionarily as a newer brain region, particularly present in primates (Cavanna and Trimble, 2006). The precuneus was also reported to be involved in self-imagery, representation of the mental self and autobiographical memory (Cavanna and Trimble, 2006). Furthermore, the precuneus was found to be involved in the evaluation of risks and benefits when establishing good reputations (Watanabe et al., 2014). Another study reported evidence on the role of the precuneus in the integration of both visuospatial information and self in the context of navigation within personal space (Freton et al., 2014). Cavanna and Trimble (2006) summarized the function of the precuneus as a richly connected multimodal associative area that belongs to a neural network, subserving awareness and producing a conscious self-percept. Regarding the activation of the precuneus in our current study, self-relevance and a link to self-representation appears to be a relevant common denominator, with a higher signaling associated with healthier food particularly in the anatomic subdivision of the cognitive/associative central area of the precuneus (Margulies et al., 2009). This area has connections to the prefrontal cortex, BA 10, 46, 8, and also to the dorsal thalamus including the lateral pulvinar, pretectal area, and superior colliculi, thus being connected with very early visual processing that is also related to emotion processing (Tamietto and de Gelder, 2010).

### Gender Differences

Several studies on food processing in the central nervous system have reported gender differences. Killgore and Yurgelun-Todd (2010) showed that women, when compared to men, had significantly greater activation to high-caloric foods within dorsolateral, ventrolateral, and ventromedial prefrontal cortex, middle/posterior cingulate, and insular brain regions. They concluded that when viewing high-calorie food images, women appear to be more responsive than men within cortical regions involved in behavioral control and self-referential cognition. Frank et al. (2010) revealed that satiation seems to influence the processing of food pictures differently in men and women in areas such as the MPFC and fusiform gyrus. On the other hand, Grabenhorst et al. (2013) did not find gender differences concerning taste preference and health-based decision variables. Geliebter et al. (2013) found obese men and women exert different brain activation toward high versus low calorie food in fed and hunger states, comprising prefrontal and subcortical areas such as the caudate. The only difference we found was stronger midbrain activation toward evaluating more healthy food items in women compared to men. One might consider a very deeply rooted healthiness signal in midbrain regions in women reflecting a primordial emotion, even though this remains speculation.

Reflecting on limitations, we have to consider the experimental condition in which the task was restricted to evaluating healthiness without the implementation of another cognitive control condition in order to assess specificity for this aspect. However, we attempted to differentiate between high and low healthiness, with the active cognitive requirement to reflect on healthiness, so that both conditions served as a control for each other with the general basis of health estimation.

## Conclusion

Differential signaling of perceived food healthiness is associated with activity in the DLPFC, MPFC, precuneus, amygdala, and ventral striatum. The overlap between food processing and emotion processing is obvious. Certainly, this overlap can be explained from an evolutionary perspective and one may even suggest that emotion processing might have its roots, at least in part, in food processing. Regarding possible implications for interventions toward nutrition behavior, one might propose an intentional active mental evaluation of the health value of food intended to be consumed. This might be combined with emotion regulation strategies aimed to reflect the accompanied appetitive emotions, such as incorporating a reality check and reappraisal toward unhealthy food. Actively reflecting on the health value of food may also enhance impulse control. The healthiness associated activation of areas involved in basic emotion processing, such as the amygdala, supports applying emotion regulation strategies in psychotherapeutic attempts to support healthy nutrition.

## Author Contributions

Substantial contributions to the conception or design of the work: UH, AB, MS, MD; acquisition, analysis, or interpretation of data for the work; UH, AB, MS, MD, CK, SO, AH, SS; drafting the work (UH) or revising it critically for important intellectual content: UH, AB, MS, MD, CK, SO, AH, SS; final approval of the version to be published and agreement to be accountable for all aspects of the work in ensuring that questions related to the accuracy or integrity of any part of the work are appropriately investigated and resolved: UH, AB, MS, MD, CK, SO, AH, SS.

## Conflict of Interest Statement

The authors declare that the research was conducted in the absence of any commercial or financial relationships that could be construed as a potential conflict of interest.
